# Risk of major postoperative complications in breast reconstructive surgery with and without an acellular dermal matrix: A development of a prognostic prediction model

**DOI:** 10.1016/j.jpra.2022.04.007

**Published:** 2022-05-12

**Authors:** N.S. Hillberg, J. Hogenboom, J. Hommes, S.M.J. Van Kuijk, X.H.A. Keuter, R.R.W.J. van der Hulst

**Affiliations:** a Department of Plastic, Reconstructive and Hand Surgery, Maastricht University Medical Center, Postal box 5800, 6202 Maastricht, The Netherlands; b School for Oncology and Developmental Biology (GROW), Maastricht University Medical Center, Postal box 616, 6200 MD Maastricht, The Netherlands; c Department of Clinical Epidemiology and Medical Technology Assessment, Maastricht University Medical Center, Maastricht, The Netherlands

**Keywords:** Acellular dermal matrix, Breast implants, Breast reconstruction, Postoperative complication, Single stage, Prognostic prediction model

## Abstract

**Introduction:**

Acellular dermal matrices (ADM) have been suggested to allow for different approaches and reduce the risk of postoperative complications in implant-based breast surgery. Surgeons seem to embrace ADMs around the world, although a lack of consistent evidence regarding the factors that increase the risk of major postoperative complications remains.

**Purpose:**

To develop and internally validate a model to predict the risk of a major postoperative complication in breast reconstructive surgery with and without an ADM.

**Methodology:**

The DBIR is an opt-out registry that holds characteristics of all breast implant surgeries in the Netherlands since 2015. Using a literature-driven preselection of predictors, multivariable mixed-effects logistic regression modelling was used to develop the prediction model.

**Results:**

A total of 2939 breasts were eligible, of which 11% underwent an ADM-assisted procedure (single-stage or two-stage). However, 31% underwent a two-stage procedure (with or without the use of ADM). Of all breasts, 10.2% developed a major postoperative complication. Age (OR 1.01), delayed timing (OR 0.71), and two-stage technique (OR 4.46) were associated with the outcome.

**Conclusion:**

The data suggest that ADM use was not associated with a major postoperative complication, while two-stage reconstructions were strongly associated with an increased risk of major complications. Despite these findings, ADMs are not as popular in the Netherlands as in the USA. The predictive capabilities of the developed model are mediocre to poor, but because of the above findings, we believe that the role of the two-stage technique as a golden standard should be put up for debate.

## Introduction

Approximately 200,000 women aged 20 to 70 years old had a breast implant in the Netherlands in 2015;^1^^,^^2^ therefore, breast implant surgery can be considered a common procedure, and figures indicate that it has not lost its popularity, despite international implant file (debates).^3^ However, breast implant surgery is not free of risk, and numerous (postoperative) complications can occur with varying incidences depending on patient characteristics, surgical technique, and implant of choice.^4^, ^5^, ^6^

Amongst the abundance of available breast reconstruction techniques, the implant is often placed underneath the pectoralis muscle. However, the pocket that should accommodate this is initially not large enough for definitive implant insertion. Therefore, traditionally, the procedure consists of two stages. The volume of the pocket is increased by the use of tissue expanders (TE), which, by stepwise stretching, allows for subpectoral implant placement in a second procedure. In contrast, single-stage breast reconstruction (SSBR) does not use a TE and often disrupts the structure of the pectoral muscle to allow for subpectoral implantation or, if possible, a subcutaneous position of the implant. Despite suggestions that a two-stage breast reconstruction surgery (TSBR) yields fewer complications in comparison with SSBR,^4^^,^^7^^,^^8^ it is evident that a SSBR is less burdensome seen from both a financial as well as a patient perspective. The introduction of the acellular dermal matrix (ADM) was thought to increase the success rate of mostly SSBR by augmenting the subpectoral pocket, as illustrated by the literature.^4^^,^^7^

ADMs were first used for breast reconstruction in 2005, where Alloderm^Ⓡ^ was mentioned in SSBR after mastectomy.^9^ In 2007, the first description of the use of Alloderm^Ⓡ^ in TSBR appeared.^10^ The use of ADMs in breast reconstruction has become common over the years, and different types of ADM have entered the market. An ADM is a piece of connective tissue in which only the extracellular matrix is still present; serving as a scaffold for adjacent tissues to extend its (a)cellular composition^11^ to dimensions that the desired implant requires. ADMs are developed from human or animal skin; all ADMs have their own prices and guidelines for storage and preparation. Alloderm^Ⓡ^ is probably the most familiar product to most plastic surgeons and is made of human cadaveric split-thickness skin graft. Also, Dermamatrix^Ⓡ^, FlexHD^Ⓡ^, AlloMax™, and DermaACELL™ are made of human dermis and are used for submuscular breast reconstruction.^12^ Strattice™ and Permacol™ are made from porcine dermis and are also used in TSBR and SSBR for submuscular reconstruction. A large randomized controlled trial about the use of Strattice™ in SSBR published in the Lancet had a lot of interest in the plastic surgeons working in the Netherlands.^13^ Braxon^Ⓡ^, also made of porcine dermis, has a different use compared to the other ADMs. Braxon^Ⓡ^ is a pre-shaped porcine ADM designed for prepectoral breast reconstruction to embrace the breast implant fully on top of the pectoralis muscle. A recently published multicentre study from the UK showed satisfactory surgical outcomes with the Braxon^Ⓡ^.^14^

Nevertheless, the evidence of the effect of using an ADM on postoperative complications is indecisive and can be interpreted as contradicting.^7^^,^^15^^,^^16^ Large-scale studies and the disclosure of well-designed registries in (single-stage or two-stage) breast implant surgery in combination with an ADM are lacking. In addition to the use of an ADM, other characteristics may be associated with postoperative complications leading to reoperation.

Prediction models are used to predict medical outcomes based on trends and patterns in available data. For example, they may identify risk factors for postoperative complications for treatment or operation (such as breast reconstruction by TSBR or SSBR). Prediction models can be helpful in the decision-making process by complementing clinicians' own clinical judgment with evidence-based analyses. Personalized medicine is growing in importance and is becoming more and more possible through this combination. By developing a reliable prediction model, counselling patients in their treatment options can be simplified and more tailor-made for the specific patient.^17^^,^^18^

Therefore, this study aimed to analyse the association between ADM and major postoperative complications and develop and internally validate a prognostic model to predict the risk of major postoperative complications (major signifying the need for revision) of breast reconstruction with and without the use of an ADM using the characteristics taken up into the Dutch Breast Implant Registry (DBIR).

## Materials and Patients

### Source of data

In the Netherlands, a registry has been set up for breast implants in 2014, namely the DBIR. Since 2015, this registry has been collecting information on all patients undergoing breast implant surgery in the Netherlands.^3^ The DBIR is an opt-out quality and implant registry which documents a collection of characteristics of the patient, the surgical procedures, and the device (i.e., an implant or a TE) of all implantation, explantation, and revision surgeries in the Netherlands. Opt-out signifies that there is no necessity of informed consent to register a patient in this registry, whereas informed consent is assumed in the willingness of the individual to undergo the procedure ^14^. The collection and disclosure of data are in line with local regulations. The professional association of plastic surgeons in the Netherlands obliges all plastic surgeons to register all patients in the registry unless a patient provides a written objection not to be registered, which is endorsed by the national health inspectorate.^3^^,^^5^^,^^19^, ^20^, ^21^ Approximately 95% of the hospitals and 74% of the private practices participate in the Netherlands and have steadily been increasing since the introduction in 2015.^3^^,^^19^ As a result of this opt-out system, the selection bias is minimized.

### Patients

Patients that underwent implant-based breast reconstruction between the 1 January 2015 and 31 December 2018 and were registered in the DBIR were included. This includes implant-based breast reconstruction after receiving a mastectomy due to breast cancer, a prophylactic mastectomy, a benign breast condition, or congenital deformities.^5^^,^^6^ Breast reconstruction was performed by a single-stage procedure with or without the use of an ADM or a two-stage procedure whether or not ADM assisted. Exclusion criteria consisted of breasts that underwent an implant-based procedure before registration; breasts that received an (first) implant in the year 2018 were excluded due to incomplete follow-up (less than 12 months); second surgery in the TSBR group not completed with sufficient follow-up; procedures with the use of an ADM in a procedure other than the first.

### Outcome

We defined a major postoperative complication as any complication leading to revision surgery in the first twelve months after surgery. A major postoperative complication can have numerous indications resulting from either patient, surgery, or implant-related complications. An overview of complications that may lead to reoperation and that were included in the DBIR is provided in Table 1. In the DBIR, all complications are assessed or diagnosed before and during the explantation or revision surgery. Consecutive major postoperative complications were not studied (i.e., haematoma after revision surgery).

**Table 1:** Summation of the indications that were defined as a possible outcome

Patient-related	Surgery-related	Device-related
└ Capsular contracture ^I^	└ Flap problems	└ Device rupture
└ Breast cancer	└ Skin scarring	└ Device deflation
└ BIA-ALCL ^II^	└ Skin necrosis	└ Silicone extravasation
└ ASIA syndrome ^III^	└ Deep wound infection	└ Device malposition
└ Breast pain	└ Seroma or haematoma	
└ Asymmetry		
└ Dissatisfaction		

Summation of indications that underly a major complication and are therefore considered as a possible outcome.

I: it is measured on the four-grade Baker scale^43^;

II: breast implant-associated anaplastic large cell lymphoma;

III: autoimmune syndrome induced by adjuvants.

### Predictors

As the number of variables collected in the DBIR is too large for implementation in a prediction model, a preselection of predictors was made based on the available literature^22^, ^23^, ^24^ and expertise; of which, an overview is provided in Table 2. All predictors were assessed by a clinician during the preoperative consultation and the first surgical procedure.^25^ In contrast to the outcome, the predictors were registered as dichotomous, categorical, or continuous, depending on the measurement level of the predictor, as is described in Table 2; the measurement unit and categories can also be found in this overview.

**Table 2:** Summation of the preselection of predictors.

Patient-related	Surgery-related	Device-related
└ Age ^φ^ in years	**└ Indication**^ψ^ as being either for mastectomy post-cancer, prophylactic mastectomy, benign breast condition, or congenital deformity	**└ Device shape ^ω^** as shaped or round
└ ASA classification ^*, ψ^ ranging from 1 to 4		**└ Device texture** ω as textured (other) or smooth
└ Radiotherapy ^ω^ as yes or no	**└ Timing ^ω^ (delay after mastectomy)** recorded as immediate or delayed	
	**└ Surgical technique (stage) ^ω^** recorded as single or two-stage	
	**└ Plane**^ψ^ as being either subglandular, subfascial, subflap, subcutaneous, subpectoral, or dual plane	
	**└ ADM/Mesh use ^ω^** as yes or no	

Summation of the preselection of predictors that were identified by the literature and expertise. ω (omega) indicating a dichotomous variable, ψ (psi) indicating a categorical variable, and φ (phi) indicating a continuous variable. Asterisk (*): American Society of Anesthesiologists physical status classification.

### Missing data

Incomplete cases were imputed using stochastic regression imputation using the fully conditional specification to minimize the likelihood of bias compared to complete case analysis as well as to prevent the loss of statistical precision. The imputed values were drawn using predictive mean matching. Cases with incomplete data in a primary determinant (i.e., surgical stage or ADM-use) were not imputed and were excluded from the dataset.

### Statistical analysis

Ideally, there should be at least 10 to 50 events available per candidate variable (EPV) whilst developing a prediction model;^26^ for techniques such as stepwise backward elimination, 50 EPV may be most suitable.^27^ The association between the preselected predictors and the occurrence of a major postoperative complication was analysed using multivariable mixed-effects logistic regression modelling to account for the clustering of observations within patients (i.e., the occurrence of bilateral procedures); to do so, a random intercept on the patient level was included in the model.^22^^,^^28^ Backward stepwise elimination was then used to arrive at a more parsimonious model, elimination was performed with an α of 0.2 to prevent early deletion of potentially important predictors. The discriminative ability and calibration of the model were computed to quantify model performance. The discriminative ability was determined using the area under the receiver operating characteristic (ROC), or AUC.^28^, ^29^, ^30^ Model calibration was evaluated by plotting deciles of the predicted probability against observed frequencies. Internal validation was then used to assess optimism and adjust for overfitting.^31^ This was performed by drawing 1,000 bootstrap samples from the original data which were then used to repeat all modelling steps. The acquired shrinkage factor allowed for the computation of the shrunk coefficients to counteract the overfitting of the model. In addition, the optimism in the AUC found by this procedure was subtracted from the apparent AUC.^32^^,^^33^ The study was reported according to the Transparent reporting of a multivariable prediction model for individual prognosis or diagnosis (TRIPOD) guidelines.^34^

## Results

### Patient demographics

**Figure 1:**
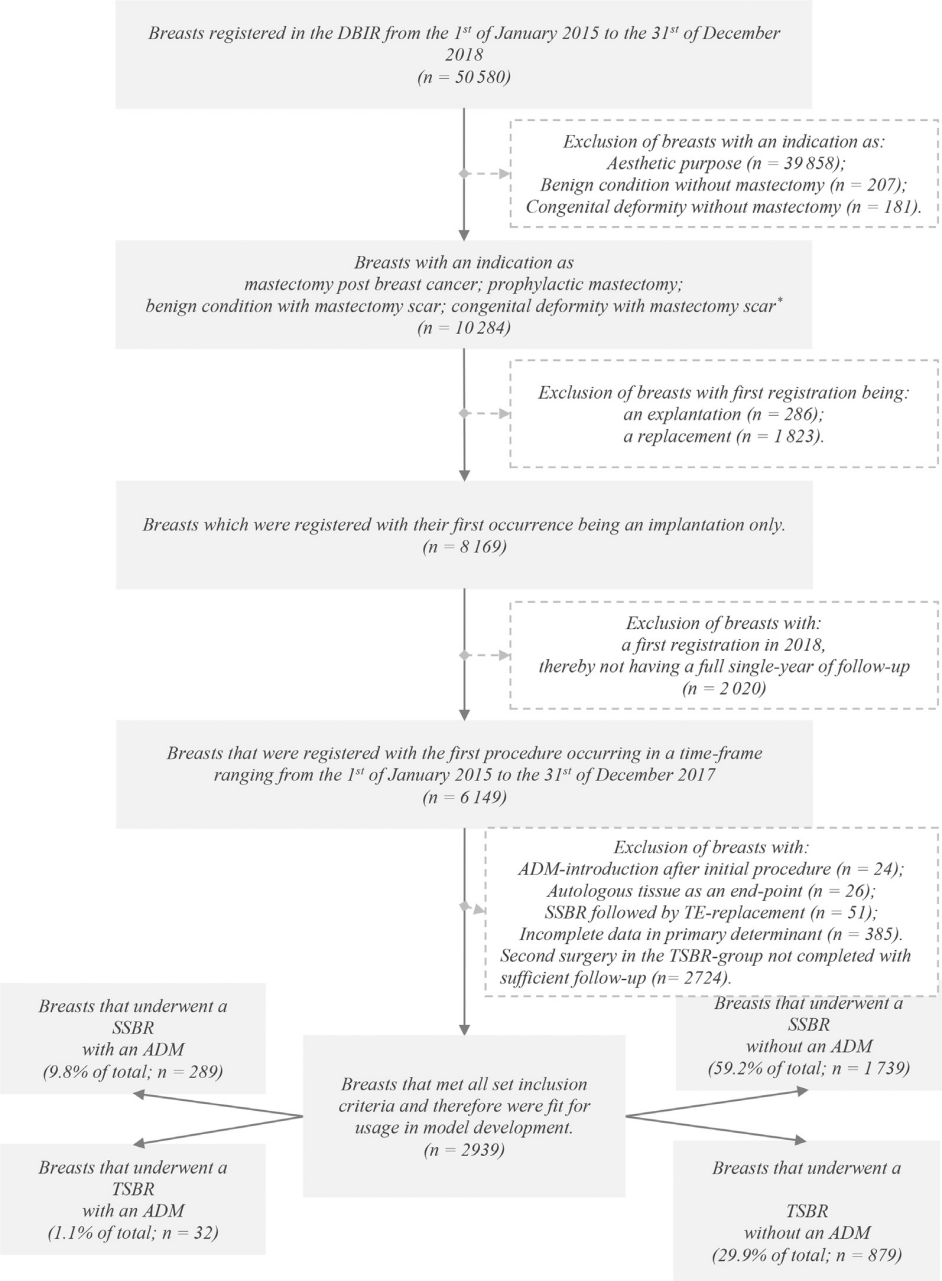

The DBIR disclosed patient, surgical and device characteristics of 50,580 breasts of 28,125 patients that underwent implant-based procedures between the 1 January 2015 and 31 December 2018. A total of 2939 breasts of 2281 patients met the inclusion criteria, as is visualized in Figure 1. The majority of reconstructions were not ADM assisted. In total, 9.8% of breasts underwent a SSBR and 1.1% a TSBR with ADM assistance.

The mean age of all included patients was 49 years old. In this sample, 85% underwent breast reconstruction post-cancer treatment, and 81% of all procedures took place immediately after mastectomy. Of all devices used, 91% were anatomically shaped, and 1% had a smooth texture. The demographics of all preselected predictors are described for each domain, per stratum, as shown in Table 3. The completeness of data within this selection of breasts differed significantly amongst the predictors, ranging from 0.1% to 24% missing with a median of 2.6%. An overview of the distribution of the major postoperative complications within the selected sample is found in Table 4. In 10% (n = 300) of the breasts, a major complication occurred. The proportion of complications found in each group varied from 4.5–5.0 % in the SSBR group to 22–31% in the TSBR group. As shown in Table 4, capsular contraction and asymmetry were the most common major complications (14% and 13%, respectively) in the overall sample. None of the major complications was due to Breast implant-associated anaplastic large cell lymphoma (BIA-ACL) or silicone extravasation. Major complications in the overall sample due to infection occurred in 5.3% of the breasts. Breast pain, flap problems, skin scarring, skin necrosis, seroma, haematoma, device rupture, or Autoimmune Syndrome Induced by Adjuvants (ASIA) syndrome were found in 5% or less of the major complications.

**Table 3:** Sample demographics.

	With ADM		Without ADM		Overall sample
Surgical technique (stage)	SSBR	TSBR	SSBR	TSBR	
Overall sample - Patients	213	22	1378	696	2281
Overall sample - Breasts	289	32	1739	879	2939
Patient characteristics					
Laterality					
Right	66	7	489	273	835
Left	73	8	514	247	842
Bilateral	150	17	736	359	1262
Intervention					
Insertion	293	32	1765	927	3017
Replacement	3	37	74	871	985
└ Unplanned	13	10	87	190	300
└ Planned	0	12	0	272	284
Explantation	5	0	18	5	28
Time to event in days as median	87	165^1^ | 164^2^	94	193^1^ | 231^2^	196
Absolute deviation	85	26^1^ | 4^2^	115	68^1^ | 143^2^	115
Age in years as mean	46.48	45.09	48.37	49.62	48.52
Standard deviation	11.48	10.50	11.84	11.51	11.72
ASA classification					
Category 1	56% (161)	901% (29)	70% (1224)	66% (581)	6% (1995)
Category 2	42% (121)	9.4% (3)	26% (446)	31% (276)	29% (846)
Category 3	2.4% (7)	0% (0)	2.8% (48)	1.7% (15)	2.4% (70)
Category 4	0% (0)	0% (0)	1.2% (21)	0.8% (7)	1% (28)
Radiotherapy	2.1% (6)	6.3%^2^	13% (227)	9.6% (84)	11% (319)
	Surgical characteristics				
Indication					
Mastectomy – Cancer	75% (218)	72% (23)	86% (1496)	85% (750)	85% (2487)
Mastectomy – prophylactic	1.4% (4)	0% (0)	0.7% (12)	0.7% (6)	0.7% (22)
Benign breast condition	23% (67)	28% (9)	13% (230)	14% (123)	15% (429)
Congenital deformity	0% (0)	0% (0)	0.1% (1)	0% (0)	0% (1)
Timing					
Immediate	9% (280)	100% (32)	80% (1382)	77% (672)	81% (2366)
Delayed	3.1% (9)	0% (0)	21% (357)	23.5% (207)	20% (573)
Incision site					
Inframammary fold	8.7% (25)	16% (5)	16% (286)	11% (93)	14% (409)
Mastectomy scar (general)	44% (127)	75% (24)	49% (849)	72% (634)	56% (1634)
Mastectomy scar (nipple-sparing)	0% (0)	0% (0)	0% (0)	0% (0)	0% (0)
Axillary	0% (0)	0% (0)	1.6% (28)	1.1% (10)	1.3% (38)
Areolar	39% (113)	6.3% (2)	12% (212)	7.2% (63)	13% (390)
Latissimus dorsi	1% (3)	3.1% (1)	9% (156)	5.6% (49)	7.1% (209)
Other	7.3% (21)	0% (0)	12% (208)	3.4% (30)	8.8% (259)
Plane					
Subglandular	1% (3)	0% (0)	5.6% (97)	1.3% (11)	3.8% (111)
Subfascial	2.1% (6)	6.3% (2)	2.8% (49)	0.9% (8)	2.2% (65)
Subflap	0.7% (2)	0% (0)	11% (182)	7.5% (66)	8.5% (250)
Subcutaneous	3.5% (10)	0% (0)	5.4% (94)	0.7% (6)	3.7% (110)
Subpectoral	58% (168)	16% (5)	41% (711)	66% (577)	50% (1461)
Dual plane	35% (100)	78% (25)	35% (606)	24% (211)	32% (942)
	Device characteristics				
Shape					
Round	2.1% (6)	3.1% (1)	11% (192)	6% (53)	8.6% (252)
Shaped or anatomical	98% (283)	97% (31)	89% (1547)	94% (826)	91% (2687)
Texture					
Textured - other	100% (289)	100% (32)	99% (1720)	99% (872)	99% (2913)
Smooth	0% (0)	0% (0)	1.1% (19)	0.8% (7)	0.9% (26)

Sample demographics with the occurrence of a certain characteristic per group of interest. With, where applicable, in brackets the number of breasts that underly the observed value in per cent

1 time to event due to tissue expander

2 time to event due to definite implant

**Table 4:** Overview of the distribution of major complications.

	With ADM		Without ADM		Overall sample
Surgical technique (stage)	SSBR	TSBR	SSBR	TSBR	
Overall sample	289	32	1739	879	2939
Any major complication	4.5% (13)	31% (10)	5% (87)	22% (190)	10% (300)
Capsular contracture	0% (0)	100% (10)	3.4% (3)	16% (30)	14% (43)
Breast cancer reoccurrence	0% (0)	0% (0)	5.7% (5)	5.8% (11)	5.3% (16)
BIA-ALCL	0% (0)	0% (0)	0% (0)	0% (0)	0% (0)
ASIA syndrome	0% (0)	0% (0)	1.1% (1)	0.5% (1)	0.7% (2)
Breast pain	7.7% (1)	0% (0)	3.4% (3)	1.1% (2)	2% (6)
Asymmetry	0% (0)	0% (0)	9.2% (8)	17% (32)	13% (40)
Dissatisfaction	0% (0)	0% (0)	4.6% (4)	7.4% (14)	6% (18)
Flap problems	7.7% (1)	30% (3)	3.4% (3)	1.6% (3)	3.3% (10)
Skin scarring	0% (0)	0% (0)	4.6% (4)	3.2% (6)	3.3% (10)
Skin necrosis	39% (5)	0% (0)	9.2% (8)	1.1% (2)	5% (15)
Deep wound infection	31% (4)	0% (0)	10.% (9)	1.6% (3)	5.3% (16)
Seroma or haematoma	15% (2)	0% (0)	3.4% (3)	2.6% (5)	3.3% (10)
Device rupture	0% (0)	0% (0)	3.4% (3)	3.7% (7)	3.3% (10)
Silicone extravasation	0% (0)	0% (0)	0% (0)	0% (0)	0% (0)
Device deflation	0% (0)	0% (0)	3.4% (3)	7.4% (14)	5.7% (17)
Device malposition	0% (0)	0% (0)	12% (10)	9.5% (18)	9.3% (28)

Overview of the distribution of major complications which necessitated revision amongst breasts within 1 year after the initial procedure. Note that the proportional incidence is relative to the total number of complications in the group of interest. With, in brackets, the number of breasts that underly the observed value in per cent.

In the ADM-assisted reconstructions, all patients requiring revision surgery in the TSBR group had to undergo revision due to capsular contracture. One of these patients had flap problems in addition to capsular contracture. In two of those patients, a third procedure was required because of flap problems that occurred after the revision surgery, whereas in the ADM-assisted SSBR-group skin necrosis and deep wound infections caused the most major complications (39% and 31%, respectively). In the non-ADM-assisted breast reconstructions, most major complications in the TSBR-group occurred due to asymmetry and capsular contraction (17% and 16%), whereas in the SSBR-group device malposition and deep wound infection were the most frequent noted major complications.

### Model specification

For clinical interpretation in combination with the lack of observations in specific data, the information provided by ASA classification, indication, and incision site was reduced using clustering of the responses. A more parsimonious model (i.e., post-backwards stepwise elimination) is found in Table 5, illustrating that the predictor's age, timing, and stage were independently associated with the occurrence of a major postoperative complication. Internal validation yielded a shrinkage factor of 0.9. The coefficients of the model were multiplied by this constant to arrive at the shrunk coefficients.

**Table 5:** Overview of the model characteristics of a model containing both surgical techniques

SSBR and TSBR				
	Coefficient	Odds ratio (95% CI)	P-value	Shrunk coefficient
Intercept	-2.699			-2.4291
	Patient characteristics			
Age in years	0.010	1.01 (1.00 to 1.02)	0.062	0.009
	Surgical characteristics			
Timing				
Immediate	Reference			
Delayed	-0.347	0.71 (0.52 to 0.96)	0.027	-0.3123
Stage				
SSBR	Reference			
TSBR	1.495	4.46 (3.47 to 5.74)	< 0.001	1.3455

Overview of the predictors and their characteristics that were found to be of significant influence on the outcome of major postoperative complication in a model which contained both single as dual-stage procedures. The model was shaped with 17 candidate predictors using 300 events in 2939 observations. Note that in the procedure of backward stepwise elimination, an α of 0.2 was used to prevent overfitting.

### Model performance

**Figure 2:**
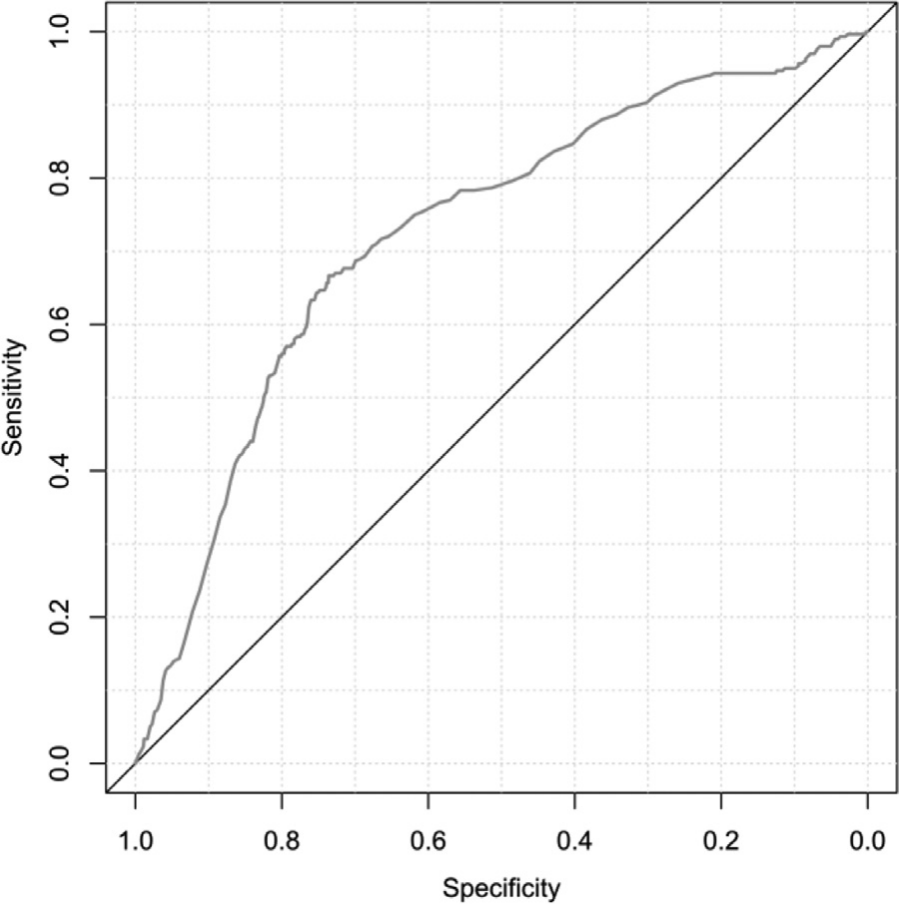

**Figure 3:**
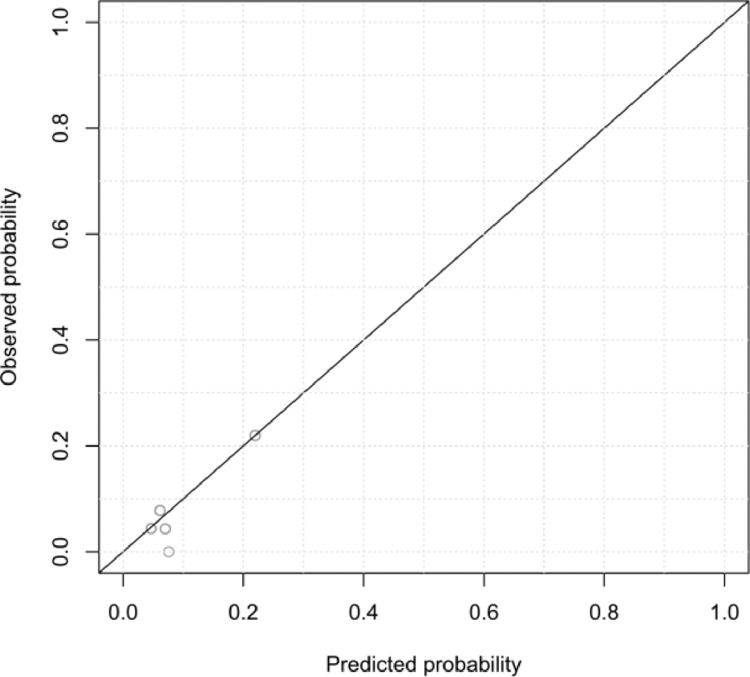

The performance of the models in terms of discriminative ability was found to be mediocre to poor. As illustrated by the ROC curve found in Figure 2, the AUC of the ROC curve was 0.725. Overall, predicted probabilities corresponded well to observed frequencies in all models are depicted by the deciles portrayed in Figure 3. The overall probabilities found in this figure can be considered low, which is a result of the relatively low odds associated with a major postoperative complication in our sample.

To avoid an optimistic predictive ability of the model, we corrected the model for this optimism. The optimism of the AUC was found to be 0.016, resulting in an optimism-corrected AUC of 0.709. In general, an AUC of 0.5 suggests no discrimination, 0.7 to 0.8 is considered acceptable, 0.8 to 0.9 is considered excellent, and more than 0.9 is considered outstanding.

## Discussion

In the field of plastic and reconstructive surgery, innovation is crucial. For such innovation, it is most desirable to contribute to a reduction in costs, on the one hand, but also improve the result and reduce the risk of complications on the other. Most of the evidence regarding postoperative complications is based on retrospective views or case series analysing different kinds of complications in uses of different kinds of ADM.^15^ In this study, we tried to create a model to predict the risk of major postoperative complications (i.e., the need for revision) in breast reconstructive surgery with and without an ADM.

We did not find ADM use to be associated with major postoperative complications in the prediction model on group level. This is in contrast to previous findings of recent studies conducted in the Netherlands^7^^,^^22^ but in line with studies conducted elsewhere.^4^^,^^15^^,^^16^^,^^35^^,^^36^ A possible explanation could be that the registered patients who had surgery after ADMs were used more in practice than in the first studies applied. Or that, for example, in the Netherlands, ADMs are only used by believers and thus experienced users in comparison with previous years in which ADMs might have been used more often. In addition, it is also possible that the ADMs used at this moment have changed. Still, these data suggest that single-stage procedures have fewer complications and ADMs might not be needed for these single-stage procedures. Future studies should, thereby, focus on the safety and aetiology of SSBR in comparison with TSBR with and without the use of different kinds of ADMs.^37^

In the Netherlands, there is no clear guideline on when to opt for an ADM. The use depends on the hospital, but especially on the plastic surgeon. Outside of the Netherlands, some surgeons only use ADMs in cases they perceive as high-risk cases (smoking, high BMI, comorbidities, (neo)adjuvant breast radiation, desire for large implant volume, etc.), but this certainly is not the case in the Netherlands.

In the Netherlands, there can be various reasons why a surgeon chooses to use an ADM or not. A history of radiotherapy on the breast, or not wanting to place the prosthesis under the muscle can be a reason to opt for an ADM. Some surgeons only use an ADM in the context of a SSBR, instead of a TSBR without an ADM. In general, the skin flaps in the Netherlands are perceived to be quite thin compared to other countries. It is, therefore, not standard practice to perform a TSBR if a lower quality of the skin flaps is observed, in these cases, no direct reconstruction is usually done at all. In the Netherlands, the large RCT about Strattice^Ⓡ^ is particularly popular, with as a result that many surgeons are not very keen on the use of ADMs in SSBR.^13^ With our study, we hope to show a different view on the use of ADMs and SSBR.

### Interpretation

In assessing the crude incidence of major complications in implant-based breast reconstruction in our dataset, it is evident that there is a substantial difference in comparing the major postoperative complication rate of the SSBR data to the TSBR data. TSBR has a strong association with a major postoperative complications (OR 4.46). However, this high OR is likely caused by more factors, which we, unfortunately, could not include in the analysis as they are not included in the DBIR dataset. It is conceivable that TSBR procedures were only performed in certain groups that, for example, had fewer comorbidities, such as a high BMI, desire for large implant volume, and who were active smokers. These factors were added recently to the DBIR and could, therefore, be added to the model in the feature.

Striking is the difference in the types of complications occurring within the different methods of breast reconstruction. Comparing SSBR with and without the use of ADM, in the ADM-assisted reconstructions skin necroses, deep wound infections and seroma or haematoma evidently occurred in a higher percentage of the breasts than in the SSBR without the use of an ADM. Although these percentages in our data were higher than those in the literature, our model showed that ADM use is not significantly associated with major postoperative complications. This is consistent with the literature.^36^^,^^38^ Device malposition and asymmetry were only seen in the reconstructions without the use of an ADM, which could suggest that ADMs provide better prevention against the malposition of the prostheses and provide better symmetry.

In addition, delayed timing is associated with decreased odds for a major complication (OR 0.71), which is not consistent with previous suggestions in the literature.^22^

As the odds of the included predictors to induce a major postoperative complication are relatively low, all developed prediction models display mediocre to poor discrimination of cases. Nonetheless, the models do fit the observed frequencies.

### Limitations

The DBIR is a relatively young registry, and factors are still actively added, such as in September 2017.^5^ As a result, risk factors that were added later in the registry such as smoking status, body mass index, and implant volume were not yet suitable for admission in the prediction model due to the lack of statistical power.^22^^,^^24^ While recent studies suggest that these factors may play a major role in the risk of postoperative complications in SSBR.^39^^,^^40^ In addition, there is insufficient information on the types of ADM that were used, as this factor was also added to the DBIR in September 2017. Resulting in a sample size unsuitable for analysis. Moreover, the DBIR being a young registry, it is sensible to assume that information bias as a result of incorrect entries is present, despite the efforts that were made to minimalize this. In addition, centre and surgeon information was not disclosed due to privacy concerns, prohibiting any insight into the influence of clustering of centres and surgical experience, despite their potential relevance.^41^^,^^42^ It is debatable whether postoperative breast pain and dissatisfaction can be considered a suitable outcome definition due to their subjective nature. However, assuming that it is likely that best efforts were made to prevent reoperation, these events were still considered to be cases as they pose to be relevant complications from a clinical perspective.

### Implications

The usefulness of the developed prediction model in terms of clinical utility can be considered to be mediocre to poor. We hypothesize that the inclusion of risk factors such as smoking status, body mass index, and implant volume in combination with a larger TSBR data can yield a more interesting result. Nonetheless, the lack of clinical utility does not imply that the results do not provide any clinically interesting findings.

First and foremost, within this non-randomized sample, ADM use was not as widely used in the Netherlands as in the USA. More importantly, ADM use was not associated with a major postoperative complication as was suggested by many studies. In contrast, we observed that there is a strong association between TSBR and revision surgeries. In terms of future clinical research, this raises the question of whether the traditional TSBR, which is considered to be the golden standard in the Netherlands, is truly the golden standard (with and also without ADM use).

## Conclusion

ADM use was not associated with an increased risk of major postoperative complications. In contrast, we observed a strong association between TSBR and major postoperative complications, which raises the question of whether the traditional TSBR should remain the golden standard for long. The usefulness of the developed prediction model in terms of clinical utility can be considered to be mediocre to poor. The inclusion of more risk factors in the DBIR database is necessary to yield a more interesting result.

## Declaration of Competing Interest

The listed authors declare to have no conflicts of interest or anything to disclose that has the potential to have introduced inappropriate bias into the presented manuscript.

## References

[1] de Boer M, van Leeuwen FE, Hauptmann M, Overbeek LIH, de Boer JP, Hijmering NJ (2018). Breast Implants and the Risk of Anaplastic Large-Cell Lymphoma in the Breast. JAMA Oncology.

[2] CBS (2019).

[3] Spronk PER, Becherer BE, Hommes J, Keuter XHA, Young-Afat DA, Hoornweg MJ (2019). How to improve patient safety and quality of care in breast implant surgery? First outcomes from the Dutch Breast Implant Registry (2015-2017). Journal of Plastic, Reconstructive & Aesthetic Surgery.

[4] Potter S, Browning D, Savović J, Holcombe C, Blazeby JM. (2015). Systematic review and critical appraisal of the impact of acellular dermal matrix use on the outcomes of implant-based breast reconstruction. British Journal of Surgery.

[5] Becherer BE. Dutch Breast Implant Registry (DBIR) Annual report 2018. 2019. 35679436

[6] Becherer BE. Dutch Breast Implant Registry (DBIR) Annual report 2015-2017. 2018. 35679436

[7] Hillberg NS, Ferdinandus PI, Dikmans REG, Winkens B, Hommes J, van der Hulst RRWJ. (2018). Is single-stage implant-based breast reconstruction (SSBR) with an acellular matrix safe?: Strattice or Meso Biomatrix(R) in SSBR. European Journal of Plastic Surgery.

[8] Dikmans RE, Negenborn VL, Bouman MB, Winters HA, Twisk JW, Ruhe PQ (2017). Two-stage implant-based breast reconstruction compared with immediate one-stage implant-based breast reconstruction augmented with an acellular dermal matrix: an open-label, phase 4, multicentre, randomised, controlled trial. The Lancet Oncology.

[9] Breuing KH, Warren SM. (2005). Immediate bilateral breast reconstruction with implants and inferolateral AlloDerm slings. Ann Plast Surg.

[10] Bindingnavele V, Gaon M, Ota KS, Kulber DA, Lee DJ. (2007). Use of acellular cadaveric dermis and tissue expansion in postmastectomy breast reconstruction. J Plast Reconstr Aesthet Surg.

[11] Boháč M, Danišovič Ľ, Koller J, Dragúňová J, Varga I. (2018). What happens to an acellular dermal matrix after implantation in the human body? A histological and electron microscopic study. European Journal of Histochemistry.

[12] Cheng A, Saint-Cyr M. (2012). Comparison of different ADM materials in breast surgery. Clin Plast Surg.

[13] Dikmans RE, Negenborn VL, Bouman MB, Winters HA, Twisk JW, Ruhé PQ (2017). Two-stage implant-based breast reconstruction compared with immediate one-stage implant-based breast reconstruction augmented with an acellular dermal matrix: an open-label, phase 4, multicentre, randomised, controlled trial. Lancet Oncol.

[14] Chandarana M, Harries S. (2020). Multicentre study of prepectoral breast reconstruction using acellular dermal matrix. BJS Open.

[15] Hallberg H, Rafnsdottir S, Selvaggi G, Strandell A, Samuelsson O, Stadig I (2018). Benefits and risks with acellular dermal matrix (ADM) and mesh support in immediate breast reconstruction: a systematic review and meta-analysis. Journal of plastic surgery and hand surgery.

[16] Stein MJ, Chung A, Arnaout A, Ghaedi B, Ghumman A, Zhang T (2020). Complication rates of acellular dermal matrix in immediate breast reconstruction with radiation: A single-institution retrospective comparison study. Journal of Plastic, Reconstructive & Aesthetic Surgery.

[17] Vogenberg FR. (2009). Predictive and prognostic models: implications for healthcare decision-making in a modern recession. Am Health Drug Benefits.

[18] van der Ploeg T. Prediction of Medical Outcomes with Modern Modelling Techniques. 2017.

[19] Rakhorst HA, Mureau MAM, Cooter RD, McNeil J, van Hooff M, van der Hulst RRWJ (2017). The new opt-out Dutch National Breast Implant Registry - Lessons learnt from the road to implementation. Journal of Plastic, Reconstructive and Aesthetic Surgery.

[20] (2018). Burgerlijk wetboek 7. Stat..

[21] (2020). Uitvoeringswet Algemene verordening gegevensbescherming. Stat.

[22] Negenborn VL, Dikmans REG, Bouman MB, Winters HAH, Twisk JWR, Ruhe PQ (2018). Predictors of complications after direct-to-implant breast reconstruction with an acellular dermal matrix from a multicentre randomized clinical trial. British Journal of Surgery.

[23] Wang C, Luan J, Panayi AC, Orgill DP, Xin M. (2018). Complications in breast augmentation with textured versus smooth breast implants: a systematic review protocol. BMJ Open.

[24] Potter S, Conroy EJ, Cutress RI, Williamson PR, Whisker L, Thrush S (2019). Short-term safety outcomes of mastectomy and immediate implant-based breast reconstruction with and without mesh (iBRA): a multicentre, prospective cohort study. The Lancet Oncology.

[25] Bargon CA, Becherer BE, Young-Afat D, van Bommel ACM, Hommes J, Hoornweg MJ (2020). The National Dutch Breast Implant Registry: user-reported experiences and importance. European Journal of Plastic Surgery.

[26] Wynants L, Collins GS, Van Calster B. (2017). Key steps and common pitfalls in developing and validating risk models. BJOG: An International Journal of Obstetrics and Gynaecology.

[27] Wynants L, Bouwmeester W, Moons KG, Moerbeek M, Timmerman D, Van Huffel S (2015). A simulation study of sample size demonstrated the importance of the number of events per variable to develop prediction models in clustered data. Journal of Clinical Epidemiology.

[28] Vergeldt TF, van Kuijk SM, Notten KJ, Kluivers KB, Weemhoff M. (2016). Anatomical Cystocele Recurrence: Development and Internal Validation of a Prediction Model. Obstetrics and Gynecology.

[29] Schoorel EN, Melman S, van Kuijk SM, Grobman WA, Kwee A, Mol BW (2014). Predicting successful intended vaginal delivery after previous caesarean section: external validation of two predictive models in a Dutch nationwide registration-based cohort with a high intended vaginal delivery rate. BJOG: An International Journal of Obstetrics and Gynaecology.

[30] Robin X, Turck N, Hainard A, Tiberti N, Lisacek F, Sanchez JC (2011). pROC: an open-source package for R and S+ to analyze and compare ROC curves. BMC Bioinformatics.

[31] Peduzzi P, Concato J, Kemper E, Holford TR, Feinstein AR. (1996). A simulation study of the number of events per variable in logistic regression analysis. Journal of Clinical Epidemiology.

[32] Steyerberg EW, Harrell FE, Borsboom GJ, Eijkemans M, Vergouwe Y, Habbema JDF (2001). Internal validation of predictive models: efficiency of some procedures for logistic regression analysis. Journal of clinical epidemiology.

[33] Harrell FE. rms: Regression modeling strategies. 2020.

[34] Moons KG, Altman DG, Reitsma JB, Ioannidis JP, Macaskill P, Steyerberg EW (2015). Transparent Reporting of a multivariable prediction model for Individual Prognosis or Diagnosis (TRIPOD): explanation and elaboration. Annals of internal medicine.

[35] Woo KJ, Park JW, Mun GH, Pyon JK, Jeon BJ, Bang SI. (2017). Does the Use of Acellular Dermal Matrix Increase Postoperative Complications of the First-Stage Reconstruction of Immediate Expander-Implant Breast Reconstruction: A Matched Cohort Study. Annals of plastic surgery.

[36] Sorkin M, Qi J, Kim HM, Hamill JB, Kozlow JH, Pusic AL (2017). Acellular Dermal Matrix in Immediate Expander/Implant Breast Reconstruction: A Multicenter Assessment of Risks and Benefits. Plast Reconstr Surg.

[37] Becherer BE, al. e. Article in press. 2020.

[38] Hunsicker LM, Ashikari AY, Berry C, Koch RM, Salzberg CA. (2017). Short-Term Complications Associated With Acellular Dermal Matrix-Assisted Direct-to-Implant Breast Reconstruction. Ann Plast Surg.

[39] Knight HJ, Musgrove JJ, Youssef MMG, Ferguson DJ, Olsen SB, Tillett RL. (2020). Significantly reducing implant loss rates in immediate implant-based breast reconstruction: A protocol and completed audit of quality assurance. J Plast Reconstr Aesthet Surg.

[40] Lardi AM, Ho-Asjoe M, Mohanna PN, Farhadi J. (2014). Immediate breast reconstruction with acellular dermal matrix: factors affecting outcome. J Plast Reconstr Aesthet Surg.

[41] Colwell AS, Damjanovic B, Zahedi B, Medford-Davis L, Hertl C, Austen WG (2011). Retrospective review of 331 consecutive immediate single-stage implant reconstructions with acellular dermal matrix: indications, complications, trends, and costs. Journal of Plastic and Reconstructive Surgery.

[42] Wynants L, Vergouwe Y, Van Huffel S, Timmerman D, Van Calster BJSmimr (2018). Does ignoring clustering in multicenter data influence the performance of prediction models?. A simulation study.

[43] Baker JL. (1975).

